# Suicidal Thoughts and Behaviors Among Swedish Suicide-Bereaved Women: Increased Risk Associated With the Loss of a Child, Feelings of Guilt and Shame, and Perceived Avoidance From Family Members

**DOI:** 10.3389/fpsyg.2020.01113

**Published:** 2020-06-05

**Authors:** Michael Westerlund, Sebastian Hökby, Gergö Hadlaczky

**Affiliations:** ^1^Department of Media Studies, Stockholm University, Stockholm, Sweden; ^2^National Centre for Suicide Research and Prevention, Centre for Health Economics, Informatics and Health Services Research, Stockholm Health Care Services, Stockholm, Sweden; ^3^National Centre for Suicide Research and Prevention, Department of Learning, Informatics, Management and Ethics, Karolinska Institutet, Stockholm, Sweden

**Keywords:** suicidal thoughts and behaviors, bereavement, child bereavement, social avoidance, guilt, shame, postvention, women’s health

## Abstract

Previous studies have shown that suicide-bereaved individuals may suffer increased risk of suicidal thoughts and behaviors (STBs) due to traumatic grief. In this paper, we present the self-reported rate of STB among Swedish suicide-bereaved women (*N* = 293). Data was collected in a cross-sectional anonymous survey on the homepages of Sweden’s leading suicide survivor organization, SPES. We used logistic regression to evaluate risks (of any STB event) related to losing a child compared to other relatives and the experience of social avoidance from family members, as well as feelings of shame and guilt. The self-reported rate of suicidal thoughts, plans, and attempts was 60, 24, and 5 percent, respectively, considerably higher than in the general population. Results showed that all of the investigated variables were independent risk factors for STB (ORs ranged between 1.29 and 2.69). Women who had both lost a child and experienced family avoidance reported the highest STB rate (87.5%), and we found an interaction effect between these two risk factors (OR = 3.45; 95% CI = 1.05–11.32) that was related to self-reported shame. It is concluded that perceived responsibility for someone else’s suicide, and the social avoidance associated with it, may play an important role for suicide survivors and should be targeted by postvention activities.

## Introduction

In Sweden, suicide is one of the most common single causes of death. With a population of 10 million people, approximately 1,500 individuals die by suicide every year (National centre for suicide research and prevention, 2019). Every death to suicide has a ripple effect and is estimated to deeply affect at least 6 to 14 relatives and close friends (Clark and Goldney, 2000; Jones and Meier, 2011; Jordan and McIntosh, 2011). Looking beyond the immediate group of bereaved family members and close friends, up to 135 individuals are in some sense affected by a single suicide (Cerel et al., 2018).

Various studies have pointed to the linkage between suicide bereavement and severe psychosocial stress and ill-health (e.g., Wilcox et al., 2010; Bolton et al., 2013; Heeke et al., 2019), and that people bereaved by suicide are at severe risk of suicidal behavior themselves (e.g., Diekstra and Garnefski, 1995; Crosby and Sacks, 2002; Runeson and Åsberg, 2003; Jordan, 2008; Erlangsen and Pitman, 2017). In a recent study, Erlangsen et al. (2017) showed that suicide-bereaved spouses have a six to eight times higher risk of completed suicide compared to the general population and also a significantly increased risk compared to individuals bereaved by other reasons (e.g., accidents and natural causes).

Bereavement after suicide seems to differ from other forms of grief (due to “other causes of death”) regarding certain characteristics. Especially strong feelings of guilt, shame, stigmatization, social rejection, and isolation have been put forward as such characteristics (e.g., Bailley et al., 1999; Jordan, 2001; Harwood et al., 2002; Ratnarajah and Maple, 2011; Bell et al., 2012). Shame and stigma have historically, culturally, and religiously surrounded the suicide subject *per se* but also many of the underlying causes of suicide, such as psychiatric illnesses, drug abuse, and social problems (Westerlund, 2018). Guilt and blameworthiness may be due to that suicide-bereaved people in part see themselves as responsible for their close one’s death (Jordan, 2001) and may feel that they should have recognized the signs and prevented the suicide (Lester, 2012).

Further, the social and cultural taboo and stigma surrounding suicide (Joiner, 2005: 6) may prevent the grief from being socially sanctioned, thereby causing bereaved individuals to withdraw and isolate themselves from their sociocultural context (Bailley et al., 1999; Jordan, 2001; Sveen and Walby, 2008; Feigelman et al., 2009; Peters et al., 2016). The taboo and stigma may also lead other people to avoid social contact with grieving suicide-bereaved individuals (Wagner et al., 2005). McMenamy et al. (2008) reported moderate-to-high levels of guilt among three quarters of suicide-bereaved subjects, and moderate-to-high levels of shame or stigma among one third. Moreover, a majority of the suicide-bereaved subjects in this study had severe difficulties in social contacts, especially with regards to communicating about suicide and sharing their grief with family members. This process of “disenfranchised grief” (Doka, 1989) can lead to an incapacity to accept the loss and create strong feelings of hopelessness and purposelessness about one’s life and future (Neimeyer et al., 2009). The unresolved, traumatic, and prolonged grief, which has also been labeled as “complicated grief” (Stroebe et al., 2001; Latham and Prigerson, 2004; Mitchell et al., 2005; Tal Young et al., 2012), may lead to adverse psychosocial reactions and suicidal behavior (Hollander, 2001; Crosby and Sacks, 2002; Bell et al., 2012).

Other studies have also indicated that social isolation and avoidance—and related psychosocial factors such as loneliness, alienation, and lack of social support—could pose a serious risk for suicidal ideation, planning, and acts (Feigelman et al., 2008; King and Merchant, 2008; McMenamy et al., 2008; van Wijngaarden et al., 2014; McKinnon et al., 2016; Stickley and Koyanagi, 2016; Beutel et al., 2017). In a recent narrative review of 40 studies on suicide and social isolation (primarily systematic reviews, meta-analyses, narrative reviews, and original studies on larger samples), Calati et al. (2019) concluded that a vast majority of studies reported a positive association between social isolation and suicidal outcomes. The authors also concluded that both the objective dimension of social isolation (living/being alone) and the subjective dimension (feeling alone/loneliness) were related to suicidal thoughts and behavior.

The prevalence of self-reported suicidal thoughts and attempts among Swedish women is 15 and 5 percent, respectively (Public Health Agency of Sweden, 2018), but the exact risk among Swedish suicide-bereaved women has hitherto been unknown, and few risk factors have been established in a Swedish context. However, some studies imply that grief processes may be particularly difficult for parents whom are losing their children to suicide (e.g., Hollander, 2001; Jordan, 2001; Bailey et al., 2015). A possible hypothesis is that both society and mothers themselves perceive a greater sense of responsibility toward their children than they do toward parents, partners, or friends. If this were the case, their own sense of failing to take responsibility could turn into feelings of guilt and shame after their child’s suicide, which in turn could add an additional layer of risk toward their own suicidality. At the same time, society’s view of the mothers’ responsibility may also exacerbate the complicated grief if it leads to greater social avoidance and a higher degree of “disenfranchisement,” especially if the mother is already experiencing feelings of guilt and shame.

In this paper, we investigate (1) the prevalence of suicidal thoughts and behaviors (STB) among Swedish suicide-bereaved women, (2) the suicide risk related to losing a child compared to losing other kin, and (3) if such an elevated suicide risk can be explained by perceived social avoidance from family members and feelings of guilt and shame.

## Methods

### Study Design

An anonymous cross-sectional web-based survey was constructed and addressed to individuals (over 18 years) who had lost a relative, a close friend, or a significant other to suicide. The survey was announced and made accessible to users on the official website and Facebook groups of Sweden’s leading suicide survivor organization, SPES^1^. Data was collected and stored on a secure server between March 18 and September 04 in 2016 and allowed only one submission per IP address. Permission was granted from the administrator of the SPES website and Facebook groups. Ethical approval for the study was granted by the Regional Ethical Review Board in Stockholm 2016-02-11. A more detailed account of the design and methods has been described elsewhere (Westerlund, 2018).

### Materials

The survey contained single item questions to measure gender, age, suicidal thoughts, plans, and attempts, the relationship/kinship to the person who had died by suicide, the perceived avoidance from family members, and feelings of guilt and shame. All participants (100%) also reported the time (number of years) that had elapsed since the death event (<1, 1–2, 2–3, 3–4, 4–5, 5–6, 6–10, or >10 years). Avoidance from family members was measured by a single item on a three-point scale (1 = Never; 2 = Sometimes; 3 = A lot). Feelings of guilt and shame was measured by single items on a five-point scale (1 = Not at all; 5 = Very much). The other items were Yes or No questions. All questions were phrased in such a way that they referred to psychological experiences occurring as a consequence of being bereaved by suicide; either soon after the event or during a process of prolonged grief [e.g., “Have you experienced that other people (in your family) have been clearly avoidant, unappreciative, or unhelpful with regards to your sorrow and pain, after the suicide of your relative/friend?”].

### Statistical Analysis

A total of 327 responses were collected. Due to a very low percentage of males, we excluded participants who reported male gender (*n* = 31) or had missing data on gender (*n* = 3), to increase the likelihood of representativeness for females. Limited statistical power precluded analysis of suicide attempts alone (see Table 1). Therefore, suicidal thoughts, plans, and attempts were indexed into a single binary measure of suicidality (“Any STB”). The measures of guilt and shame were treated as continuous variables. All other items were originally categorical or ordinal variables and were transformed (dichotomized) to simplify the interpretation of the results. All analyses were performed in IBM SPSS version 25 with a significance level of 0.05 (two-tailed). Bivariate associations were estimated using Chi-square tests, Pearson correlations, and independent samples *t*-tests.

**TABLE 1:** Rate (%) of self-reported suicidal thoughts, plans, and attempts (STB) in the total sample and separately for child-bereaved participants.

Group	Thoughts	Plans	Attempts	Any STB
All women (*N* = 293)	60.1%	23.5%	4.8%	60.8%
Missing data (% of 293)	1.7%	6.1%	6.5%	2%
Child loss group (*n* = 116)	69.8%	34.5%	4.3%	71.6%
Loss of other kin (*n* = 177)	53.7%	16.4%	5.1%	53.7%
Group difference (Pearson Chi-square)	$\chi_{\left(1\right)}^{2}$ = 8.74; *P* = 0.003	$\chi_{\left(1\right)}^{2}$ = 14.67; *P* < 0.001	$\chi_{\left(1\right)}^{2}$ = 0.04; *P* = 0.837	$\chi_{\left(1\right)}^{2}$ = 8.40; *P* = 0.004

In the main analysis, we modeled STB using a three-step hierarchical logistic regression, always controlling for the effect of age (*N* = 275, after listwise exclusion of missing cases). We modeled the effects of losing a child versus losing other kin (partner, parent, sibling, other family relative, friend, other relations), the perceived avoidance from family members (dichotomized as “Not at all” vs. “To some degree” or “A lot”), and feelings of shame and guilt (continuous scores; 1–5). Importantly, we also modeled the interaction effect between child loss and family avoidance (Child loss × Family avoidance) and examined if such an effect was contingent on the level of guilt and shame reported by participants. The first step in the regression included age, child loss, and family avoidance; the interaction term was included in the second step; and guilt and shame were included in the third step. This specific sequence of entering predictors to the model enabled us to examine whether any interaction effect was contingent guilt and shame.

## Results

### Univariate and Bivariate Analyses

In the total sample of bereaved women (*N* = 293), the frequency of suicidal thoughts, plans, and attempts was about 60, 23, and 5 percent, respectively, and the index of any SBT was 61 percent (Table 1). Forty percent of the sample reported having lost a child to suicide, and Chi-square analyses showed that these women more commonly (relative to the other 60%) reported suicidal thoughts and plans, but not attempts. The risk of any STB was also significantly higher in the child loss group (Table 1). The amount of time (number of years) that had elapsed since the death event was typically a few years (median value = 2–3 years), but this variable was not statistically associated with STB [*t*_(285)_ = −1,623; *P* = 0.078].

The mean age in the total sample was 46.8 years (SD = 13.4; range = 18–79), and the mean age was significantly higher in the child loss group [56.6 vs. 40.3 years; *t*_(290)_ = −12.58; *P* < 0.001]. About half of the sample (53.2%) reported at least some degree of family avoidance. The mean shame score was 2.35 (SD = 1.44; range = 1–5), and the mean guilt score was 3.65 (SD = 1.35; range = 1–5).

### Regression Analysis

The first model step (*P* < 0.001) indicated significant main effects of both child loss (OR = 2.69) and family avoidance (OR = 2.30), in the expected direction. The interaction term (child status × avoidance status) term was entered in the second step, but this did not significantly improve the model (*P* = 0.059). The model only improved significantly at the third step (*P* < 0.001), in which the shame and guilt variables were entered into the model. In this final model, there were no significant main effects of child loss, family avoidance, or age. However, the interaction term significantly predicted suicidal outcomes (OR = 3.45), as did shame (OR = 1.29) and guilt (OR = 1.31). Age was not significantly related to suicidal outcomes in any step of the model. The detailed test results from the regression are shown in Table 2.

**TABLE 2:** Results from binary logistic regression model: prediction of any STB (suicidal thoughts, plans, and/or attempts).

Model entry sequence	Predictors	Odds ratio (95% CI)		*P*-value
Step 1	Child status	2.69	(1.39–5.21)	0.003
	Family avoidance status	2.30	(1.37–3.85)	0.002
Step 2	Child status	1.72	(0.77–3.83)	0.186
	Family avoidance status	1.62	(0.86–3.04)	0.133
	Interaction (Child × Family avoidance status)	2.95	(0.94–9.27)	0.065
Step 3	Child status	1.55	(0.67–3.58)	0.304
	Family avoidance status	1.32	(0.68–2.57)	0.415
	Interaction (Child × Family avoidance status)	3.45	(1.05–11.32)	0.041
	Shame	1.29	(1.02–1.62)	0.032
	Guilt	1.31	(1.05–1.64)	0.016
Overall model statistics	All models controlled for the effect of age (*P* > 0.05 in all steps)			
	Step 1, Omnibus test: $\chi_{\left(3\right)}^{2}$ = 22.29; *P* < 0.001; −2 Log likelihood = 342.41; *R*${}_{\text{Nagelkerke}}^{2}$ = 0.10			
	Step 2, Omnibus test: $\chi_{\left(1\right)}^{2}$ = 3.57; *P* = 0.059; −2 Log likelihood = 338.83; *R*${}_{\text{Nagelkerke}}^{2}$ = 0.11			
	Step 3, Omnibus test: $\chi_{\left(3\right)}^{2}$ = 20.27; *P* < 0.001; −2 Log likelihood = 318.57; *R*${}_{\text{Nagelkerke}}^{2}$ = 0.20			
	(Final model Omnibus test: $\chi_{\left(6\right)}^{2}$ = 44.13; *P* < 0.001)			

### *Post hoc* Analyses

Tests of bivariate associations were performed *post hoc* to disentangle the regression analysis and increase the interpretability of its results. Mainly, the effects of shame and guilt were examined using *t*-tests (because the *P*-values were virtually the same, we here report the results under assumption of homoscedasticity, even if violated). Firstly, there was a moderately large correlation between shame and guilt (*r*_Pearson_ = 0.50; *P* < 0.001), but both were independently associated with STB in the expected direction [mean shame score difference = −0.70; *t*_(282)_ = −4.06; *P* < 0.001 (heteroscedasticity indicated); mean guilt score difference = −0.67; *t*_(280)_ = −4.18; *P* < 0.001]. Secondly, there was no significant difference in shame or guilt between the child loss group and the participants [mean shame score difference = −0.02; *P* = 0.902; mean guilt score difference = −0.25; *P* = 0.132 (heteroscedasticity indicated)]. Thirdly, the group of participants that reported family avoidance had significantly higher scores on shame while there was no such group difference in guilt [mean shame score difference = −0.58; *t*_(282)_ = −3.43; *P* = 0.001 (heteroscedasticity indicated); mean guilt score difference = −0.21; *P* = 0.185]. Fourth, a Chi-square analysis showed that perceived family avoidance was not more commonly reported in the child loss group (50.0%) compared to the other women (57.2%; *P* = 0.229). Fifth, we did not find any Pearson correlation with age (Shame: *P* = 0.298; Guilt: *P* = 0.541). Lastly, the amount of time (number of years) that had elapsed since the death event had no significant impact on the regression model (coefficient statistics: OR = 1.09; *P* = 0.139; details not shown).

**FIGURE 1:**
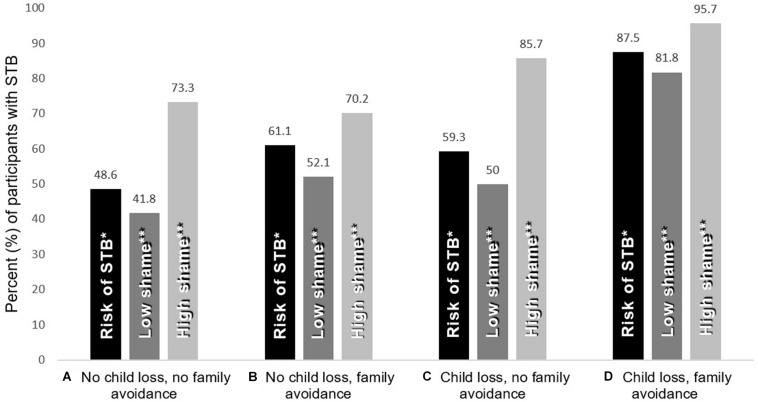
Descriptive results for participants included in the Logistic regression analysis (*N* = 275). Rate (%) of suicidal thoughts and behaviors (STB) in various subgroups, according to variations in shame. The four groups **(A–D)** represent the 2×2 combinations of child loss and family avoidance status. *The STB risk when disregarding shame all together. **Shame levels split at median (Low=1–2; High=3–5).

Together, these *post hoc* tests provide an indication of why the interaction term was not significant when shame was not included in the regression. That is, the combination of child loss and family avoidance *did* imply a multiplicative risk of STB, but this interaction was mediated by shame, which was not in itself associated with child loss. Moreover, descriptive results (shown in Figure 1) may suggest that the additive risk of child loss + family avoidance created a ceiling effect, as 87.5% of participants in this subgroup reported STB. Hence, shame had a relatively small impact in this group, but was more critical for STB in the other subgroups (especially A and C). Figure 1 shows the different STB rates in different subgroups according to shame levels (dichotomized at the median; Md = 2). However, guilt levels contributed less to the understanding of this interaction effect, as it was only directly associated with STB (and with shame). This is confirmed by the fact that the results of the regression were virtually unchanged when replicated without including guilt in the model (results not shown). Guilt is therefore not displayed in Figure 1.

## Discussion

This study aimed to investigate the rate of self-reported STB among adult Swedish women who had lost someone to suicide—with particular attention to women who had lost a child to suicide. Over half of the sample reported having suicidal thoughts (compared to 15% in the Swedish general population), and about five percent reported suicidal attempts (compared to 5% of the Swedish general population) (Public Health Agency of Sweden, 2018). Overall, these numbers were even higher in the child loss group. Due to the relatively small sample size, risk of self-selection bias, and other biases, these prevalence figures cannot be generalized. Nonetheless, they can be considered high by many standards, especially in relation to healthy populations. Considering that participants accessed the survey through a suicide survivor organization (SPES), the elevated risk of STB is not entirely unexpected. However, this is the first study, to our knowledge, to report such figures for Swedish women.

About half of the sample reported that their family, at least to some degree, had been “avoidant, unappreciative or unhelpful with regards to their sorrow and pain.” A main effect was found on STB in this group (a 2.3-fold increased risk). Higher shame scores (but not guilt scores) were also associated with both family avoidance and STB, but not child loss. Finally, we found an interaction effect between child loss and family avoidance, but only when shame scores were held constant (for participants who lost a child the relative odds increased from 2.69 to 3.45). In other words, the co-occurrence of both these risk factors implied not only an additive risk (where 87.5% reported STB) but also a multiplicative risk. This interaction was found when the high-risk women were compared to other participants with equal levels of shame. However, since shame was only associated with family avoidance but not with the type of loss (losing a child, spouse, parent, etc.), it remains unclear why this group is more vulnerable to STB in terms of shame. Moreover, guilt contributed less to our understanding of this interaction, as it was only directly associated with STB. In other words, participants who experienced both child loss and family avoidance evidenced the risk of STB regardless of whether they felt guilty or not. Although guilt and shame are often used interchangeably, the results of this study suggest that these are two somewhat different, even if related constructs (cf. Hastings et al., 2002). Further support of this is the finding that the items measuring shame and guilt only had 25% shared variance.

One possible understanding of why child-bereaved mothers are at increased risk for STB is that feelings of shame (poor self-image and self-blame) grow as a consequence of being socially avoided by family members (cf. Hollander, 2001; Grad, 2005; Bell et al., 2012; Bailey et al., 2015). This avoidance may be interpreted as a sign of rejection or punishment—even if it is not so intended—especially if one views the suicide as a sign of bad character and failure to take social responsibility (Jordan, 2001; Bell et al., 2012; Lester, 2012). Our data supports the notion that bereaved mothers have a particularly high risk of STB if they experience shame and/or if the family avoidance is associated with some factor that is unrelated to shame but still makes the grief process more challenging (e.g., loneliness). There are various signs of complicated grief that might discourage family members from expressing their sympathies, due to fear of negative emotional reactions (cf. Sveen and Walby, 2008; Feigelman et al., 2009; Peters et al., 2016). Poor knowledge and stigmatizing attitudes toward suicide and mental health problems might contribute to such social strains (cf. Wagner et al., 2005; McMenamy et al., 2008).

### Limitations of the Study

The present study has a number of limitations, such as the cross-sectional design which precludes further investigation of temporal changes. Also, the retrospective character of the study may have caused a biased reporting, and the self-selective approach affects who chooses to participate (the most obvious example is the over-representativeness of females in the sample). However, our analysis of time (years elapsed since the suicide) indicated a neglectable effect of time, producing statistically non-significant effect in all main aspects, including the regression model. The study is also largely descriptive in its nature, as the reasons behind the suicidal acts, feelings of shame, and guilt, etc., were not investigated in further depth (e.g., we did not collect qualitative data regarding these issues). Therefore, the research questions and hypothetical mechanisms that we put forth in this paper can only be answered in terms of general statistical associations. For example, the study does not provide a clear picture of *why* some participants experienced more shame or family avoidance than others. Further, the cross-sectional nature does not allow any conclusions about the causal or temporal direction between these variables. Our conclusions are thus tentative, with regards to why child-bereaved mothers are at increased risk for STB, relative to other suicide-bereaved women.

### Implications of the Study

The clinical implications of this study pertain to social support in the context of shame and self-blame. Public health-related postvention activities should aim to increase the chances of social support from close relatives, whereas psychotherapeutic interventions may target feelings of shame and guilt in an attempt to improve the grieving process. As Bell et al. (2012) have suggested, suicide-bereaved people need help and support to understand that the guilt, shame, and responsibility they feel for their relative’s death in suicide is most often overestimated. However, therapeutic, psychiatric, and other interventions are probably not equally effective during all stages of a grieving process. Regardless of the statistical risk faced by this group of bereaved women, our results indicate that informal support (such as social acceptance) from one’s family may be of similar protective importance. Hence, there is an imperative to longitudinally investigate critical stages in the grieving process, whereby psychological construct such as guilt and shame should be treated as targets of therapy. Qualitative studies may shed light on the psychological mechanism that should be targeted by clinical intervention. Public health initiatives should also be stimulated in order to identify bereaved individuals at risk of self-harm, using for example larger quantitative datasets and electronic health registers.

### Conclusion

In this study, we found a high self-reported rate of STB among Swedish suicide-bereaved women. We also found that women who had lost a child were at increased risk of those outcomes (except suicide attempts). Although the child loss group was not more prone to report avoidance from family members, the subgroup of bereaved mothers who did experience such avoidance were at especially increased risk of STB. However, we also found that feelings of guilt and shame were important predictors of STB. Shame was particularly important in mediating the interaction (i.e., the exacerbated risk) between child loss and family avoidance. Almost all (over 95%) bereaved mothers who reported both family avoidance and high levels of shame also reported STB and should be offered appropriate postvention.

## Data Availability Statement

The datasets generated for this study are available on request to the corresponding author.

## Ethics Statement

The studies involving human participants were reviewed and approved by the Regional Ethical Review Board in Stockholm. Written informed consent for participation was not required for this study in accordance with the national legislation and the institutional requirements.

## Author Contributions

MW and SH were the main contributors to the production of this manuscript, to which GH made important intellectual contributions. MW conceived of the original study, designed the questionnaire, conducted the survey, and collected the data. SH conceptualized the analytic plan and conducted the statistical analysis. GH made intellectual contributions to the analytic plan and results interpretations. MW and SH authored the manuscript to which GH made substantial contributions. All authors read and approved the final manuscript.

## Conflict of Interest

The authors declare that the research was conducted in the absence of any commercial or financial relationships that could be construed as a potential conflict of interest.

## Footnotes

1 www.spes.se
